# Genes Affect Population Growth, but the Environment Determines How

**DOI:** 10.1371/journal.pbio.0040150

**Published:** 2006-04-25

**Authors:** Richard Robinson

Why does a population in the wild grow or shrink, or remain the same over generations? Genes would seem an obvious factor, but in fact there is remarkably little evidence that genetic effects influence year-to-year population dynamics, beyond the well-recognized negative effect from inbreeding on very small populations.

This lack of evidence for a genetic effect partly reflects the difficulty of choosing which gene to study, as only a few genes may be under strong selection at any given time, and fewer still will affect population dynamics. It also partly reflects an important truth about natural selection—it may determine who survives to reproduce, but not necessarily the total number of survivors. In many cases, ecological factors such as resource abundance and natural enemies may overwhelm any slight genetic effect on population dynamics.

Finding a genetic effect, then, would require both knowing which pin in the genetic haystack to look for and having detailed knowledge of the complex and powerful environmental factors against which this effect plays out. In a new study, Ilkka Hanski and Ilik Saccheri show that variants of a sugar-metabolizing gene do indeed influence population growth in a species of butterfly, but in a complex and habitat-dependent way.

The authors studied the Glanville fritillary butterfly on the Åland Islands in Finland, where its population dynamics are well studied, and its habitat—patches of dry meadows spread across the landscape—is well mapped. They focused on the gene phosphoglucose isomerase *(Pgi)*, a key enzyme in the breakdown of sugar. The *Pgi* gene occurs in several forms, or alleles, whose proteins differ in their kinetic properties and thermal stability. These alleles have been previously linked to differences in flight metabolic rate and fecundity in this butterfly, making the gene a good candidate for observing a population effect, if there is one. The *f* and *d* alleles of *Pgi* are the most common, and previous work has shown that butterflies with either an *ff* or an *fd* genotype have a higher flight metabolic rate and are more fecund than those with a *dd* genotype.

By analyzing genotypes, population growth, and habitat area simultaneously among more than 130 small butterfly populations, the authors showed that, in small meadows, growth was highest when the *ff* or *fd* genotypes predominated, but in larger meadows, the opposite was true—these genotypes predicted a decline in numbers instead of a rise, while *dd* was favored. The effect appeared to be specific to *Pgi*, as there was no correlation with genotype for any of the six other genes.

The likely explanation for this effect, according to the authors, is related to the differences in maturation and egg laying between females bearing *f* and *d* alleles. Those with *f* alleles mature quickly and lay more eggs early on, just the strategy for exploiting a small patch, from which many butterflies risk drifting away rather quickly in their life. Those with *d* alleles mature later but also die later, allowing them to exploit a larger habitat more thoroughly. However, the authors note that this may not be the only, or even the main, reason for the genotype-habitat area effect, since *Pgi* is likely to influence many different aspects of life history.

The results of this study confirm that, under the right circumstances, intraspecific genetic variation can influence population growth. But they also make an important point about fitness. A major goal of evolutionary physiology is to understand the selective advantage of the traits found in a population, and it is often tempting in this pursuit to assume there is a single “best” genotype. This study provides a strong counterargument against such one-size-fits-all models of evolution, pointing out that in the ecological theater, which script plays best is a function of exactly which stage you are on.

## 

**Figure:**
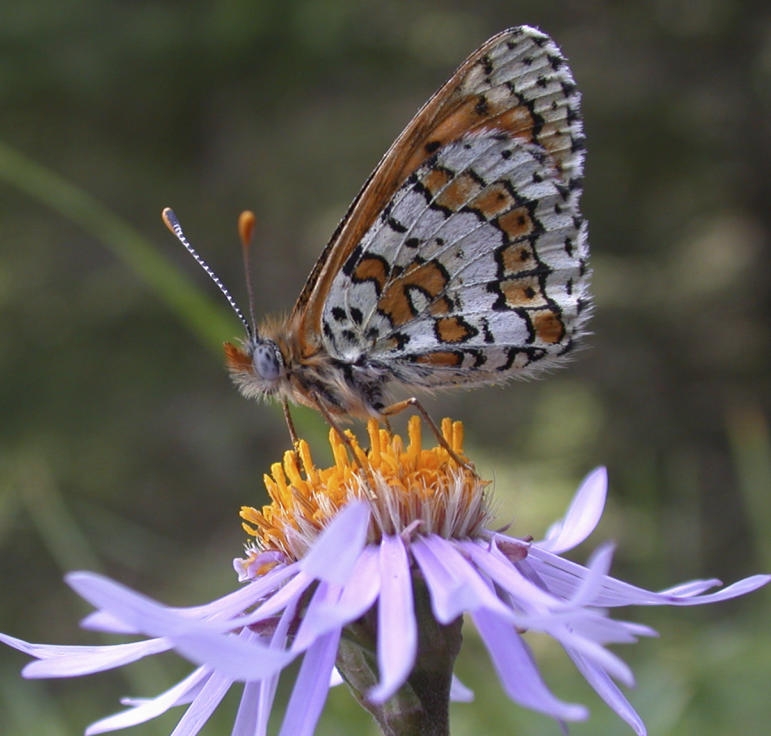
The Glanville fritillary butterfly.

